# Prevalence and Management of Oral Intake Restrictions in Critically Ill Patients: Insights from a Multicenter Point Prevalence Study

**DOI:** 10.1007/s00455-024-10772-5

**Published:** 2024-10-21

**Authors:** Takashi Hongo, Tetsuya Yumoto, Keibun Liu, Kensuke Nakamura, Akira Kawauchi, Takefumi Tsunemitsu, Nobuto Nakanishi, Atsunori Nakao, Hiromichi Naito, Oral Intake in I. C. U. study group

**Affiliations:** 1 Department of Emergency, Critical Care, and Disaster Medicine, Okayama University Graduate School of Medicine, Dentistry, and Pharmaceutical Sciences, 2-5-1 Shikata, Okayama, 700-8558 Japan; 2 Non-Profit Organization ICU Collaboration Network (ICON), 2-15-13 Hongo, Bunkyo-Ku, Tokyo, 113-0033 Japan; 3 Critical Care Research Group, The Prince Charles Hospital, 627 Rode Rd, Chermside, QLD 4032 Australia; 4 Institute for Molecular Bioscience, The University of Queensland, 306 Carmody Rd, St Lucia, QLD 4067 Australia; 5 Yokohama City University School of Medicine, 3-9 Fukuura, Kanazawaku, Yokohama, 236-0004 Japan; 6 Department of Critical Care and Emergency Medicine, Japanese Red Cross Maebashi Hospital, 389-1, Asakura-Machi, Maebashi-Shi, Gunma 371-0811 Japan; 7 Department of Preventive Services, Kyoto University School of Public Health, Yoshida-Honmachi, Kyoto Sakyo-Ku, Kyoto, 606-8501 Japan; 8 Division of Disaster and Emergency Medicine, Department of Surgery Related, Kobe University Graduate School of Medicine, 7-5-2 Kusunoki, Chuo-Ward, Kobe, 650-0017 Japan

**Keywords:** SLT, Dysphagia, Oral intake, Swallowing rehabilitation, PICS

## Abstract

Oral intake restrictions due to dysphagia in the intensive care unit (ICU) can increase morbidity, mortality, and negatively impact quality of life. The current oral intake practice and clinical management strategies for addressing dysphagia in the ICU are not well-defined. This study aimed to elucidate the clinical practices surrounding oral intake restrictions due to dysphagia and its management strategies in the ICU. A multicenter, prospective, cross-sectional, 2-day point prevalence study was conducted in Japan. Relevant data on the clinical circumstances surrounding oral intake practice and the implementation of strategies to prevent dysphagia for patients admitted to the ICU on November 1, 2023, and December 1, 2023, were collected. The primary outcome was the prevalence of oral intake restrictions in patients, defined by a Functional Oral Intake Scale score of less than 7 among eligible patients for oral intake. Out of 326 participants, 187 were eligible for the final analysis after excluding 139 patients who were not eligible for oral intake, primarily due to tracheal intubation. Among those eligible, 69.0% (129/187) encountered oral intake restrictions. About 52.4% (98/187) of patients underwent swallowing screenings; 36.7% (36/98) of these were suspected of having dysphagia. Compensatory and behavioral swallowing rehabilitation were provided to 21.9% (41/187) and 10.6% (20/187) of patients, respectively, from ICU admission to the survey date. Only 27.4% (14/51) of post-extubation and 9.3% (3/32) of post-stroke patients received swallowing rehabilitation. Notably, no ICUs had dedicated speech and language therapists, and most (85.7%, 18/21) lacked established swallowing rehabilitation protocols. This 2-point prevalence survey study revealed that oral intake restrictions due to dysphagia are common in ICUs, but few patients are screened for swallowing issues or receive rehabilitation. More clinical studies are needed to develop effective protocols for identifying and managing dysphagia, including screenings and rehabilitation in the ICU.

**Supplementary Information:**

The online version contains supplementary material available at 10.1007/s00455-024-10772-5.

## Introduction

Critically ill patients admitted to the intensive care unit (ICU) frequently face oral intake restrictions due to various reasons [1]. These can include not only intubation, but also decreased level of consciousness resulting from primary neurological impairment or secondary to sedative drugs, intolerance due to respiratory or hemodynamic instability, facial injury or major surgery around the face or neck, and gastrointestinal intolerance such as post-gastrointestinal surgery or ileus. Additionally, substantial psychological disability or loss of appetite can also contribute to oral intake restrictions [2, 3]. Among these factors, dysphagia, or the inability to swallow food or liquid, is a major and recognized critical issue in the ICU. Conditions such as post-extubation dysphagia and post-stroke dysphagia are prevalent, affecting approximately 40% of patients who have been mechanically ventilated and 80% of acute stroke patients, respectively [4–7]. Dysphagia is recognized to be associated with increased morbidity and mortality. Management of dysphagia may warrant oral intake restrictions, which can lead to delay the resumption of oral intake and lead to malnutrition [8, 9]. Consequently, these issues can prolong the length of stay in the ICU and/or hospital, reduce the likelihood of discharge to home, and increase healthcare costs [10, 11]. Despite their crucial importance, the standard practices for managing dysphagia in Japanese ICUs remain unknown.

Post-intensive care syndrome (PICS) is defined by the presence of worsening impairments in physical, cognitive, or mental health status arising following critical illness. It impacts the long-term prognosis and quality of life in critically ill patients [12]. Oral intake restrictions attributed to dysphagia can be considered one of the risk factors for PICS [12]. Despite the significant impact of ICU-developed dysphagia on patient well-being and quality of life, it is inadequately covered in the relevant guidelines, likely due to a lack of high-quality evidence on dysphagia assessment (how, when, and by whom) and oral intake restriction management in the ICU [13, 14]. This scarcity of evidence stems from insufficient data on both the recognition of this issue and existing practice patterns, highlighting the urgent need to bridge these gaps for enhanced understanding and intervention.

Consequently, the aim of this study was to investigate the current practices for identifying and managing patients with oral intake restrictions, including dysphagia, in Japanese ICUs. To the best of our knowledge, this study contributes foundational data for establishing protocols aimed at improving oral intake to prevent and treat dysphagia associated with critical illness.

## Methods

### Study Design, Setting, and Ethics

This was a multicenter, prospective, cross-sectional, 2-day point prevalence study conducted in Japanese ICUs, approved by the Okayama University Ethics Committee (K2310-031). The study was spearheaded by the Japanese Society for Early Mobilization and designed in accordance with the STROBE guidelines for cross-sectional studies. In line with the Ethical Guidelines for Medical and Health Research Involving Human Subjects in Japan [15], the requirement for ethical approval at each participating institution was waived, as no participant identifiable data were collected in this study. This ethical protocol was shared with all clinicians, with ICU participation depending on their agreement after reviewing regional ethics policies.

### Survey Process

First, the study recruited participating institutions from September 8 to October 31, 2023, by sending an invitation letter to members of the Japanese Society for Early Mobilization, along with other local networks This letter provided a brief overview of the study, including a link to the website or a paper-based questionnaire that detailed the study’s objectives and ethical considerations. Upon consenting to participate, representatives from each ICU—comprising intensivists, physician, critical care nurses, speech and language therapists (SLTs), physical therapist, and registered dietitian nutritionist—were asked to submit basic information about their hospital/ICU’s demographics and background. After this information was provided, each ICU was registered as a participating site. The study then conducted 2-point prevalence surveys on Wednesday, November 1, and Friday, December 1, 2023, both at 10 a.m. Participating ICUs were asked to complete the survey and to write the same representative’s name on each one. The day before each survey date, the institution’s representatives received a URL for an online questionnaire or a paper-based questionnaire for the ICU care survey, enabling them to respond via their preferred method. To ensure the quality of the survey and consistent answers, participants were encouraged to collaborate with other healthcare professionals when completing the questionnaire.

### Survey Questionnaire

A panel of 9 intensivists collaborated to create and review a structured questionnaire. This questionnaire was developed based on the findings from previous studies and our clinical interests [11.16]. The questionnaire sought basic information on hospital/ICU demographics and detailed data on swallowing evaluation and rehabilitation practices prior to the 2-day prevalence study. Additional File 1 presents the initial survey (24 questions, estimated completion time: 20 min) covering hospital and ICU type, number of hospital and ICU beds, number of SLTs, presence of a protocol for swallowing rehabilitation, and components of swallowing screening and rehabilitation. A dedicated SLT is defined as an SLT who exclusively works in the ICU and is not assigned to other wards. The 2-point prevalence study was designed to capture patient characteristics. Additional File 2 provides a guide for representatives conducting a 2-day prevalence study (79 questions, estimated completion time: 60 min). In addition to baseline characteristics of the patients, detailed information from ICU admission to the survey date, including data on the methods and personnel involved in swallowing screening and assessing swallowing function, and details of swallowing rehabilitation, encompassing both compensatory and behavioral swallowing rehabilitation strategies. Compensatory strategies include postural adjustments to make swallowing safer, as well as diet modifications, such as adjusting the texture of food (e.g., pureeing solids or thickening liquids), to make swallowing easier and safer. Behavioral swallowing rehabilitation includes techniques such as ice massage, oral motor exercise, thermal-tactile stimulation, and salivary gland massage. Ice massage is widely used in Japan as a pre-feeding technique to induce dry swallowing, stimulate swallowing apraxia to initiate the swallow action, and as part of daily swallowing training, which involves applying an ice stick applied to the throat, base of the anterior faucial arches, base of the tongue, and posterior pharyngeal wall [17]. Swallowing function at the time of survey was collected using the Functional Oral Intake Scale (FOIS), which is a tool commonly used to measure oropharyngeal dysphagia. Scores vary from 1, indicating no oral intake, to 7, which signifies a complete oral diet without any restrictions; higher scores reflect improved swallowing ability [4]. To distinguish dysphagia from oral intake restrictions due to other reasonable medical reasons, we additionally collected information on the number of patients whose primary reasons for oral intake restrictions were categorized into five groups: respiratory instability, hemodynamic instability, altered level of consciousness, gastrointestinal issues, and others. Furthermore, we conducted an additional survey on the role of dentists in the ICU, as they are considered to play a significant role. This survey was conducted on June 29, 2024 (Additional File 3). To ensure that the responses reflected the situation at the time of the original surveys conducted in 2023, all respondents from the initial survey were asked to provide information based on the circumstances in 2023.

### Outcomes

The primary outcome was the prevalence of oral intake restrictions in patients, defined as having a FOIS score of less than 7 among eligible patients for oral intake [18]. Eligible patients for oral intake were defined as those excluding patients who had endotracheal intubation and those with oral intake restrictions due to reasons other than dysphagia, as mentioned in the 5 categories above. Secondary outcomes included the implementation rates of dysphagia-related care among eligible patients for oral intake, such as swallowing screening, swallowing rehabilitation and diet modification, and swallowing protocol among each participating ICU. Further analyses were conducted on specific patient subgroups, namely those post-extubation and post-stroke [4, 5].

### Data Analysis

We used descriptive statistics to outline the baseline characteristics of both the participating ICUs and patients, focusing on the overall cohort, those eligible for oral intake, as well as post-extubation and post-stroke patients in a subgroup analysis. The prevalence of oral intake restrictions and practice patterns for dysphagia were estimated based on the proportion of patients eligible for oral intake in the two-day survey. Continuous variables were described using the median and interquartile ranges (IQR), whereas categorical variables were presented as numbers and percentages. Statistical analyses were performed using Stata version 18 (StataCorp LP, College Station, TX).

## Results

### Demographic and Medical Data of the Participating ICUs

A total of 21 ICUs across Japan participated in the study, with the predominant ICU type being mixed medical-surgical (95.3%, 20/21). The majority of survey were provided by intensivists, who accounting for 61.9% (13/21) of the contributions. Table 1 shows characteristics of the participating institutions.

**Table 1:** Characteristics of participating ICU in the study

	All ICUs (n = 21)
Type of hospital, n (%)	
University hospital	6 (28.6)
Community hospital	15 (71.4)
Number of hospital beds, n (%)	
0–100	1 (4.7)
101–300	0 (0.0)
301–500	10 (47.6)
501–800	8 (38.0)
800-	2 (9.5)
Number of ICU beds, n (%)	
0–5	0 (0.0)
6–10	9 (42.8)
11–20	4 (19.0)
21–30	5 (23.8)
31–40	1 (4.7)
40-	2 (9.5)
Type of ICU, n (%)	
Mixed medical/surgical	20 (95.3)
Medical	0 (0.0)
Surgical	1 (4.7)
Primary source of ICU admission, n (%)	
Emergency room	11 (52.3)
Wards	1 (4.7)
Operation room	9 (42.8)
Transfer	0 (0.0)
Responder’s professional role, n (%)	
Intensivist	13 (61.9)
Physician (Not intensivist)	2 (9.5)
Speech and language therapist	2 (9.5)
Physical therapist	2 (9.5)
Critical care nurse	1 (4.7)
Registered dietitian nutritionist	1 (4.7)

*ICU* intensive care unit

No ICUs had dedicated SLTs, and majority (85.7%, 18/21) did not have specific protocol for swallowing rehabilitation. Dysphagia screening was predominantly conducted using the water swallowing test (76.1%, 16/21), with nurses as the main screeners (85.7%, 18/21). The most frequently utilized rehabilitation techniques were ice massage (57.1%, 12/21) and oral exercises (52.3%, 11/21), primarily administered by nurses (42.8%, 9/21) and SLTs (47.6%, 10/21). Although dentists provide dental services in 61.9% (13/21) of participating ICUs, only 1 ICU (4.7%) is involved in the assessment or treatment of dysphagia. Table 2 provides further details on the characteristics of dysphagia management and swallowing rehabilitation practices in the ICUs, as obtained from the initial survey.

**Table 2:** Characteristics of swallowing rehabilitation in participating ICU

	All ICUs (n = 21)
The number of SLTs in hospital, n (%)	
0	2 (9.5)
1	0 (0.0)
2	2 (9.5)
3	4 (19.0)
4–5	7 (33.3)
6–10	5 (23.8)
Unknown	1 (4.7)
SLTs dedicated to the ICU, n (%)	
Yes	0 (0.0)
No	21 (100)
Average duration per session of swallowing rehabilitation in the ICU, n (%)	
0 min	6 (28.5)
1–5 min	2 (9.5)
6–10 min	2 (9.5)
11–15 min	3 (14.2)
16–30 min	7 (33.3)
31- min	1 (4.7)
Frequency of swallowing rehabilitation in the ICU, n (%)	
Not at all	6 (28.5)
Less than every other weekday and no weekends	2 (9.5)
Less than every other weekday and weekends	0 (0.0)
Every other weekday and no weekends	2 (9.5)
Every other weekday and weekends	2 (9.5)
Every weekday and no weekends	7 (33.3)
Every weekday and weekends	2 (9.5)
Swallowing rehabilitation protocol in ICU, n (%)	
Yes	3 (14.2)
No	18 (85.7)
Primary bedside screening tool for dysphagia, n (%)	
Water swallowing test / modified water swallowing test	16 (76.1)
Repetitive saliva swallowing test	3 (14.2)
Both	1 (4.7)
Not at all	1 (4.7)
Clinician primarily conducts screening for dysphagia, n (%)	
Nurse	18 (85.7)
SLT	2 (9.5)
Unknown	1 (4.7)
Behavioral swallowing rehabilitation in the ICU, n (%)	
Swallowing exercises	9 (42.8)
Ice massage of pharynx	12 (57.1)
Lip closure training	1 (4.7)
Oral motor exercise (exercises for the lips, tongue, jaw, and cheeks)	11 (52.3)
Thermal-tactile stimulation	1 (4.7)
Toothbrushing	9 (42.8)
Salivary gland massage	5 (23.8)
Electrical stimulation therapy	0 (0.0)
Not at all	2 (9.5)
Clinician primarily performs behavioral swallowing rehabilitation in the ICU, n (%)	
Nurse	9 (42.8)
SLT	10 (47.6)
Not at all	2 (9.5)
Clinician primarily determines dietary intake and content for patients with dysphagia in the ICU, n (%)	
Intensivist	3 (14.2)
Attending physician	4 (19.0)
Nurse	8 (38.0)
SLT	6 (28.5)
Common reasons for difficulty in continuing swallowing rehabilitation, n (%)	
Altered states of consciousness (including delirium)	15 (71.4)
Decreased oxygen levels (hypoxia)	14 (66.6)
Rapid breathing	6 (28.5)
Hemodynamic instability	5 (23.8)
Sudden arrhythmia	2 (9.5)
High risk of aspiration/choking	9 (42.8)
Gastrointestinal issues such as gastrointestinal bleeding, ileus, etc	9 (42.8)
Inability to assume a sitting position	4 (19.0)
Dentists provide dental services in ICU, n (%)	13 (61.9)
ICU treatment performed by dentist, n (%)	
Tooth extraction	8 (38.0)
Oral care	6 (28.5)
Fixed denture	5 (23.8)
Trauma treatment	5 (23.8)
Postoperative management in oral surgery	2 (9.5)
Dysphagia assessment	1 (4.7)

*ICU* intensive care unit, *SLTs* speech and language therapists, *IQR* interquartile range

### Prevalence and Eligibility for Oral Intake

Table 3 summarizes the findings from a 2-day point prevalence survey. Among the 326 participants, 124 (38.0%) were intubated, 56 (17.1%) were post-extubation, and 25 (7.6%) had a tracheostomy on the survey date at 10 a.m. A total of 32 patients (9.8%) were suffering from a stroke. Of the 326 participants, 139 were not eligible for oral intake due to endotracheal intubation (89.2%, 124/139), gastrointestinal issues (6.4%, 9/139), respiratory instability (0.7%, 1/139), hemodynamic instability (0.7%, 1/139), altered level of consciousness (0.7%, 1/139), and others factors (2.1%, 3/139).

**Table 3:** Demographics and characteristics from the 2-day point prevalence survey

	All n = 326	First survey n = 160	Second survey n = 166
Age (yr), n (%)			
0 to 19	18 (5.5)	11 (6.8)	7 (4.2)
20 to 64	86 (26.3)	40 (25.0)	46 (27.7)
65 to 74	107 (32.8)	37 (23.1)	70 (42.1)
75 or older	115 (35.2)	72 (45.0)	43 (25.9)
Sex, n (%)			
Male	192 (58.9)	84 (52.5)	108 (65.0)
Female	134 (41.1)	76 (47.5)	58 (35.0)
Patients’ status, n (%)			
Post-extubation	56 (17.1)	31 (19.3)	25 (15.0)
Tracheostomy	25 (7.6)	12 (7.5)	13 (7.8)
Post-stroke	32 (9.8)	20 (12.5)	12 (7.2)
Oral diet with no restrictions (FOIS score of 7) before ICU admission, n (%)	271 (83.1)	126 (78.7)	145 (87.3)
Oral diet status on the survey date, n (%)			
Reasonable reasons for not eligible for oral intake, n (%)	139 (42.6)	67 (41.8)	72 (43.3)
Endotracheal intubation	124 (89.2)	59 (88.0)	65 (90.2)
Respiratory instability	1 (0.7)	1 (1.4)	0 (0.0)
Hemodynamic instability	1 (0.7)	1 (1.4)	0 (0.0)
Altered level of consciousness	1 (0.7)	0 (0.0)	1 (1.3)
Gastrointestinal issues	9 (6.4)	5 (7.4)	4 (5.5)
Others	3 (2.1)	1 (1.4)	2 (2.7)
Eligibility for oral intake, n (%) ^a^	187 (57.3)	93 (58.1)	94 (56.6)
FOIS:1	174 (53.3)	81 (50.6)	93 (56.0)
Potentially eligible for oral intake among FOIS 1, n (%)	35 (18.7)	14 (15.0)	21 (22.3)
FOIS:2 or 3	37 (19.8)	23 (24.7)	14 (14.8)
FOIS:4	12 (6.4)	7 (7.5)	5 (5.3)
FOIS:5 or 6	45 (24.1)	23 (24.7)	22 (23.4)
FOIS:7	58 (31.0)	26 (27.9)	32 (34.0)
Oral intake restriction (FOIS < 7), n (%) ^b^	129 (69.0)	66 (70.9)	63 (67.0)
Swallowing screening, n (%) ^c^			
Any swallowing screening ^a^	98 (52.4)	48 (51.6)	50 (53.1)
Swallowing screening via water swallowing test or modified water swallow test ^d^	81 (82.6)	36 (75.0)	45 (90.0)
Any swallowing screening by a nurse ^d^	86 (87.8)	44 (91.7)	42 (84.0)
Dysphagia suspected patients, n (%)	41 (12.5)	15 (9.3)	26 (15.6)
Dysphagia suspected based on swallowing screening ^d^	36 (36.7)	12 (25.0)	24 (48.0)
Dysphagia suspected but swallowing screening was not performed ^e^	5 (5.6)	3 (6.8)	2 (4.4)
Swallowing function assessment, n (%) ^a^			
Assessment via VFSS or FEES	2 (1.0)	2 (2.1)	0 (0.0)
Swallowing rehabilitation, n (%)			
Compensatory swallowing rehabilitation ^a^	41 (21.9)	19 (20.4)	22 (23.4)
Compensatory swallowing rehabilitation via a SLT ^a^	22 (11.7)	10 (10.7)	12 (12.7)
Compensatory swallowing rehabilitation via a nurse ^a^	25 (13.3)	11 (11.8)	14 (14.8)
Compensatory swallowing rehabilitation for suspected dysphagia based on swallowing screening ^f^	18 (50.0)	9 (75.0)	9 (37.5)
Behavioral swallowing rehabilitation ^a^	20 (10.6)	11 (11.8)	9 (9.5)
Behavioral swallowing rehabilitation via a SLT ^a^	16 (8.5)	7 (7.5)	9 (9.5)
Behavioral swallowing rehabilitation via a nurse ^a^	9 (4.8)	8 (8.6)	1 (1.0)
Behavioral swallowing rehabilitation for suspected dysphagia based on swallowing screening ^f^	13 (36.1)	6 (50.0)	7 (29.1)

^a^ Excluding those who were endotracheally intubated and those with oral intake restrictions due to reasons other than dysphagia, primarily categorized into one of five reasons: respiratory instability, hemodynamic instability, altered level of consciousness, gastrointestinal issues, and others

^b^ Among patients who were eligible for oral intake

^c^ Conducted from ICU admission to the survey date

^d^ Among those screened

^e^ Based on subjective clinical indicators (e.g., choking, coughing). Among those who were not screened, excluding those who were endotracheally intubated and those with oral intake restrictions due to reasons other than dysphagia, primarily categorized into one of five reasons: respiratory instability, hemodynamic instability, altered level of consciousness, gastrointestinal issues, and others

^f^ Among those who were suspected dysphagia based on swallowing screening

*FEES* fiberoptic endoscopic evaluation of swallowing, *FOIS* function oral intake scale, *ICU* intensive care unit, *SLT* speech and language therapist, *VFSS* videofluoroscopic swallowing study

**Fig. 1:**
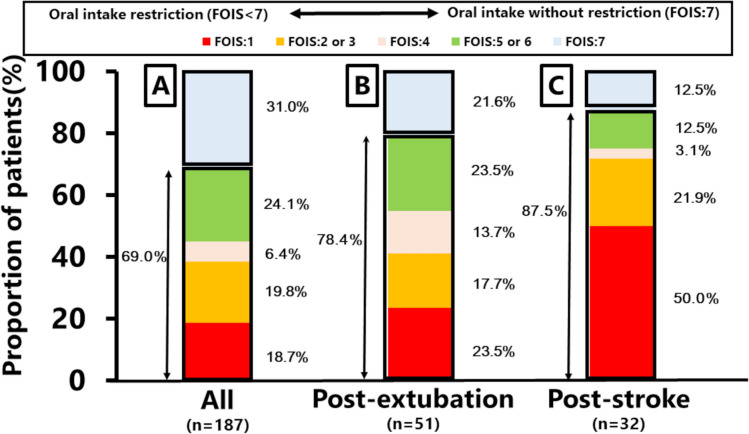
Distribution of patients’ oral intake levels based on the FOIS among eligible patients for oral intake. **A**: Overall population, **B**: Post-extubation patients, **C**: Post-stroke patients. *FOIS* function oral intake scale

Of the 187 patients eligible for oral intake after excluding these 139 patients, 69.0% (129/187) exhibited dysphagia, leading to oral intake restrictions, as defined by a FOIS score of 6 or less. The FOIS score distribution was as follows: 18.7% with FOIS = 1, 19.8% with FOIS = 2 or 3, 6.4% with FOIS = 4, and 24.1% with FOIS = 5 or 6 (Fig. 1A).

### Swallowing Assessment and Management

Of the 187 who were eligible for oral intake, 52.4% (98/187) underwent swallowing screening, predominantly using the water swallowing test or its modified version, administered by nurses (87.8%, (86/98)). Among those screened, 36.7% (36/98) were suspected of having dysphagia. Among those who were not screened, 5.6% (5/89) were suspected of having dysphagia based on clinical symptoms such as coughing or choking during meals, and the presence of aspiration pneumonia. The implementation rate of videofluoroscopic swallowing studies (VFSS) or fiberoptic endoscopic evaluation of swallowing (FEES) for swallowing assessment was only 1.0% (2/187). Compensatory and behavioral swallowing rehabilitation were provided to 21.9% (41/187) and 10.6% (20/187) of patients, respectively, from ICU admission to the survey date. Among the patients with suspected dysphagia, compensatory and behavioral rehabilitation were applied to 50.0% (18/36) and 36.1% (13/36), respectively. These interventions were primarily administered by nurses and SLTs. SLTs were involved in swallowing function assessment and swallowing rehabilitation for less than 10% of all patients.

### Post-extubation and Post-stroke Patients

Table 4 presents the demographics and characteristics of post-extubation and post-stroke patients. Of the 17 post-extubation patients with an FOIS of 1, 12 were potentially eligible for oral intake after excluding those with oral intake restrictions due to reasons other than dysphagia (n=5). In post-stroke patients, all those with an FOIS of 1 (n=16) were eligible for oral intake. The prevalence of oral intake restriction among eligible patients for oral intake was 78.4% (40/51) in post-extubation patients (Figure 1B) and 87.5% (28/32) in post-stroke patients (Figure 1C), respectively. Swallowing screening was conducted for 78.4% (40/51) of post-extubation patients and 21.8% (7/32) of post-stroke patients. Compensatory and behavioral rehabilitation were provided to 27.4% (14/51) of post-extubation patients and 9.3% (3/32) of post-stroke patients, with these interventions primarily administered by SLTs.

**Table 4:** Demographics and characteristics of post-extubation and post-stroke patients

	Post-extubation			Post-stroke		
	All (n = 56)	First survey (n = 31)	Second survey (n = 25)	All (n = 32)	First survey (n = 20)	Second survey (n = 12)
Oral diet status on the survey date, n (%)						
Reasonable reasons for oral intake restrictions other than dysphagia, n (%)						
Endotracheal intubation	N/A	N/A	N/A	0 (0.0)	0 (0.0)	0 (0.0)
Respiratory instability	1 (5.9)	1 (14.3)	0 (0.0)	0 (0.0)	0 (0.0)	0 (0.0)
Hemodynamic instability	1 (5.9)	1 (14.3)	0 (0.0)	0 (0.0)	0 (0.0)	0 (0.0)
Altered level of consciousness	0 (0.0)	0 (0.0)	0 (0.0)	0 (0.0)	0 (0.0)	0 (0.0)
Gastrointestinal issues	1 (5.9)	1 (14.3)	0 (0.0)	0 (0.0)	0 (0.0)	0 (0.0)
Others	2 (11.7)	1 (14.3)	1 (10.0)	0 (0.0)	0 (0.0)	0 (0.0)
Eligibility for oral intake, n (%) ^a^	51 (91.0)	27 (87.0)	24 (96.0)	32 (100)	20 (100)	12 (100)
FOIS:1	17 (30.3)	7 (22.5)	10 (40.0)	16 (50.0)	12 (60.0)	
Potentially eligible for oral intake among FOIS 1, n (%)	12 (70.6)	3 (42.8)	9 (90.0)	16 (50.0)	12 (60.0)	4 (33.3)
FOIS:2 or 3	9 (16.0)	5 (16.1)	4 (16.0)	7 (21.8)	1 (5.0)	6 (50.0)
FOIS:4	7 (12.5)	4 (12.9)	3 (12.0)	1 (3.1)	1 (5.0)	0 (0.0)
FOIS:5 or 6	12 (21.4)	9 (29.0)	3 (12.0)	4 (12.5)	4 (20.0)	0 (0.0)
FOIS:7	11 (19.6)	6 (19.3)	5 (20.0)	4 (12.5)	2 (10.0)	2 (16.6)
Oral intake restriction (FOIS < 7), n (%) ^b^	40 (78.4)	21 (77.7)	19 (79.1)	28 (87.5)	18 (90.0)	10 (83.3)
Swallowing screening, n (%) ^c^						
Any swallowing screening ^a^	40 (78.4)	22 (81.4)	18 (75.0)	7 (21.8)	5 (25.0)	2 (16.6)
Swallowing screening via water swallowing test or modified water swallowing test ^d^	13 (32.5)	8 (36.3)	5 (27.7)	5 (71.4)	4 (80.0)	1 (50.0)
Any swallowing screening by a nurse ^d^	35 (87.5)	18 (81.8)	17 (94.4)	5 (71.4)	3 (60.0)	2 (100)
Dysphagia suspected patients, n (%)	11 (19.6)	5 (16.1)	6 (24.0)	3 (9.3)	2 (10.0)	1 (8.3)
Dysphagia suspected based on swallowing screening ^d^	10 (25.0)	5 (22.7)	5 (27.7)	2 (28.5)	1 (20.0)	1 (50.0)
Dysphagia suspected but swallowing screening was not performed ^e^	1 (9.0)	0 (0.0)	1 (20.0)	1 (4.0)	1 (6.6)	0 (0)
Swallowing assessment, n (%) ^a^						
Assessment via VFSS or FEES	2 (3.9)	2 (7.4)	0 (0.0)	0 (0)	0 (0)	0 (0)
Swallowing rehabilitation, n (%)						
Compensatory swallowing rehabilitation ^a^	14 (27.4)	9 (33.3)	5 (20.8)	3 (9.3)	2 (10.0)	1 (8.3)
Compensatory swallowing rehabilitation via a SLT ^a^	11 (21.5)	6 (22.2)	5 (20.8)	3 (9.3)	2 (10.0)	1 (8.3)
Compensatory swallowing rehabilitation via a nurse ^a^	3 (5.8)	3 (11.1)	0 (0.0)	0 (0.0)	0 (0.0)	0 (0.0)
Compensatory swallowing rehabilitation for suspected dysphagia based on swallowing screening ^f^	10 (100)	5 (100)	5 (100)	2 (100)	1 (100)	1 (100)
Behavioral swallowing rehabilitation ^a^	14 (27.4)	8 (29.6)	6 (25.0)	3 (9.3)	2 (10.0)	1 (8.3)
Behavioral swallowing rehabilitation via a SLT ^a^	11 (21.5)	5 (18.5)	6 (25.0)	3 (9.3)	2 (10.0)	1 (8.3)
Behavioral swallowing rehabilitation via a nurse ^a^	7 (13.7)	6 (22.2)	1 (4.1)	0 (0.0)	0 (0.0)	0 (0.0)
Behavioral swallowing rehabilitation for suspected dysphagia based on swallowing screening ^f^	8 (80.0)	3 (60.0)	5 (100)	2 (100)	1 (100)	1 (100)

^a^ Excluding those who were endotracheally intubated and those with oral intake restrictions due to reasons other than dysphagia, primarily categorized into one of five reasons: respiratory instability, hemodynamic instability, altered level of consciousness, gastrointestinal issues, and others

^b^ Among patients who were eligible for oral intake

^c^ Conducted from ICU admission to the survey date

^d^ Among those screened

^e^ Based on subjective clinical indicators (e.g., choking, coughing). Among those who were not screened, excluding those who were endotracheally intubated and those with oral intake restrictions due to reasons other than dysphagia, primarily categorized into one of five reasons: respiratory instability, hemodynamic instability, altered level of consciousness, gastrointestinal issues, and others

^f^ Among those who were suspected dysphagia based on swallowing screening

*FEES* fiberoptic endoscopic evaluation of swallowing, *FOIS* function oral intake scale, *ICU* intensive care unit, *SLT* speech and language therapist, *VFSS* videofluoroscopic swallowing study

## Discussion

This point prevalence survey, conducted across 21 ICUs in Japan involving 326 patients, examined the current prevalence of oral intake restrictions and its management practices in the ICU setting. Although we could not rigorously distinguish dysphagia from oral intake restrictions due to reasonable medical reasons, the present study reveals that the overall prevalence of oral intake restrictions due to dysphagia was nearly 70% among those eligible for oral intake. Swallowing screenings were conducted for only 50% of the patients, mostly by nurses, and swallowing rehabilitation was provided to approximately 20% of the patients by nurses and SLTs. Notably, even when focusing on specific patient groups such as those post-extubation or post-stroke, the prevalence of oral intake restrictions due to dysphagia remained approximately 80%. However, a significantly smaller proportion of these patients received swallowing screenings or rehabilitation, especially among post-stroke patients.

Our study revealed that none of the ICUs had dedicated SLTs, and 85.7% lacked a specific protocol for swallowing rehabilitation. These findings are similar to or even worse than those of previous studies [16, 19, 20]. Indeed, implementing protocols can enhance swallowing function across various patient populations [21]. Furthermore, we discovered that approximately 76% of ICUs employ the water swallowing test for dysphagia assessment, primarily conducted by nurses. This rate closely matches the screening practices reported in a broader international survey [16]. Additionally, our findings suggest that gold-standard diagnostic methods such as FEES or VFSS are rarely employed [22, 23], a situation that can be attributed to a lack of resources, the absence of specialists, and logistical challenges. Although dentists in Japan provide oral care to inpatients to improve oral health in the wards [24], our study revealed that few ICUs involve dentists in the assessment and/or treatment of oral health issues, including dysphagia. Given the limited availability of SLTs and dentists in Japan [16] and the prevailing challenges, the essential role of ICU nurses in dysphagia care is emphasized. Research supports the success of nurse-implemented swallowing rehabilitation following an initial evaluation by SLTs, provided the nurses receive adequate training [25–27]. This emphasizes the importance of a collaborative approach, integrating the expertise of both SLTs and trained nursing staff to improve patient outcomes. While the specific impact of nurses’ educational backgrounds on this study is unclear, nurse-led bedside swallowing screenings likely play a critical role in identifying patients at risk of dysphagia and ensuring safe oral intake for those not at risk. Adequate education and training for nurses in bedside swallowing screenings and rehabilitation are essential for ensuring safe oral intake, early dysphagia detection, and improved swallowing function. We need to establish swallowing protocols for dysphagia that are tailored to each facility in Japan.

Although Mclntyre et al. showed 41% of critical ill patients had post-extubation dysphagia in their meta-analysis, the definition of dysphagia varied among studies [7]. More recent studies have shown that 93% of post-extubated patients exhibited dysphagia based on the FOIS, and 58.6% of them required enteral feeding. [28, 29]. Another recent study demonstrated that 87% of ICU patients referred to SLTs were diagnosed with dysphagia, and 51% couldn’t initiate oral intake [30]. Our findings are similar, with only 30% of patients able to have oral intake without restrictions, underscoring the prevalent issue of dysphagia in ICU settings. Notably, despite guidelines recommending dysphagia screening for all stroke patients to prevent post-stroke pneumonia [31], only 21.8% of post-stroke patients underwent such screenings, highlighting the urgent need to acknowledge this issue and promote necessary screenings and appropriate subsequent rehabilitation. Compared to other countries, Japan lags behind in the current management for the prevention of oral intake restrictions, including swallowing screening and rehabilitation for dysphagia [16]. This emphasizes the importance of recognizing and establishing effective practices in this critical area.

Our study has several limitations. First, oral intake can be affected by factors beyond swallowing function, such as medication effects, delirium, and gastrointestinal symptoms, making it challenging to clearly distinguish between ‘oral intake restriction’ and ‘dysphagia’ in our survey. Second, as a point prevalence study, it captures a single moment in time, limiting causality or tracking temporal changes. This snapshot may not fully represent dynamic factors such as mechanical ventilation duration, disease severity, or patient consciousness levels. Third, specific timing of the survey may not reflect post-survey dysphagia evaluations and interventions, potentially misestimating their prevalence. Forth, our observation that 5.6% of non-screened patients were suspected of having dysphagia could be an underestimation, highlighting the potential oversight of dysphagia in this population. Fifth, the 2-day point prevalence study is conducted with a 30-day interval; however, if patients are hospitalized for an extended period, repeated assessments of the same patients may occur. Finally, potential bias may have been introduced by the individuals administering the survey, which could have affected the accuracy of data collection. Additionally, the fact that not all patients had their dysphagia evaluated by SLTs further limits the accuracy of the data collected.

Despite these limitations, our study provides insight into current real-world practices for swallowing screening, assessment, and management across more than 20 ICUs in Japan. Even the most recent guidelines from The European Society for Clinical Nutrition and Metabolism mention little about dysphagia management in the ICU, with no specific guidance on screening methods or rehabilitation strategies [3]. The clear need for a collaborative, multidisciplinary approach to tackle dysphagia in ICUs highlights a critical area for future research. This involves aiming to develop and implement comprehensive care strategies to effectively manage dysphagia [21].

## Conclusions

This multicenter point prevalence survey study showed that the overall prevalence of oral intake restrictions was high, with only a minority of patients undergoing swallowing screenings, and an even smaller subset receiving swallowing rehabilitation. There is a pressing need for further clinical studies to establish effective protocols or a dysphagia care bundle aimed at better identifying patients with dysphagia and implementing appropriate management strategies in the ICU, including swallowing screenings and rehabilitation.

## Supplementary Information

Below is the link to the electronic supplementary material.

Supplementary file1 (DOCX 30 KB)—First survey questionnaire

Supplementary file2 (DOCX 28 KB)—Two-day prevalence survey questionnaire

Supplementary file3 (DOCX 20 KB)

## Data Availability

The datasets used and/or analyzed during the current study are available from the authors on reasonable request.

## Abbreviations

FOIS: Functional oral intake scale
FEES: Fiberoptic endoscopic evaluation of swallowing
ICU: Intensive care unit
IQR: Interquartile ranges
PICS: Post-intensive care syndrome
SLT: Speech and language therapist
VFSS: Videofluoroscopic swallowing studies
