# Supplementary material for: Prevalence and Management of Oral Intake Restrictions in Critically Ill Patients: Insights from a Multicenter Point Prevalence Study
Source: Dysphagia. 2024 Oct 21;40(4):747–58. doi: 10.1007/s00455-024-10772-5 (PMC12328520; doi:10.1007/s00455-024-10772-5)
Supplement: Supplementary file 1 — Supplementary file1 (DOCX 30 KB)—First survey questionnaire [file 455_2024_10772_MOESM1_ESM.docx]

**Additional File 1**

**Survey Questionnaire**

﻿ You are invited to participate in a cross-sectional survey study focusing on the care of patients with oral intake restrictions in the intensive care unit (ICU). We recently reported delayed initiation of speech and language therapy in post extubation dysphagia patients was associated with persistent dysphagia or death. Early initiation of speech and language therapy may prevent dysphagia. This survey has the potential to improve our understanding of dysphagia in ICU

This survey consists of two parts: "Basic ICU Information" and "Point Prevalence Survey”

Before conducting the Point Prevalence Survey, there are approximately 24 questions regarding the basic information of the ICU

This questionnaire is completely voluntary. No personally identifiable information about the patients will be collected. All data will be kept securely, and only aggregated data will be published. If you have any questions about the questionnaire, please feel free to contact the following:

Please review the following explanation, and if you are willing to participate in this study, check the "Agree" box at the bottom.

Important Notes:

1. If there are multiple ICUs within the hospital, please provide responses only for one ICU per respondent. If you need to provide answers for other ICUs, please change the respondent. (High Care Units (HCU) are not included in this survey.)

2. Both survey require data collection from medical records and healthcare professionals. Since this study involves gathering information from medical records and healthcare professionals, it may take more time compared to other survey studies. To ensure the quality of the survey and consistent answers, participants were encouraged to collaborate with other healthcare professionals when completing the questionnaire. Your participation is crucial to the success of the research, despite the time and effort required. We appreciate your cooperation.

Study coordinator

Takashi Hongo, MD, PhD

Department of Emergency, Critical Care, and Disaster Medicine

Faculty of Medicine, Dentistry and Pharmaceutical Sciences, Okayama University, Okayama, Japan

Email: pwup5kuf@s.okayama-u.ac.jp

Phone: 086-235-7426

**1. Are you participated in this survey?**

**a Agree**

**b Not Agree**

**2. Representative's name**

**( )**

**Section 1: Demographics**

**1. What is your professional role?**

a. intensivists

b. Physician (Not intensivists)

c. Critical care nurse

d. Speech and language therapist

e. other ( )

**2. What type is your institution certified?**

a. University hospital

b. Community hospital

c. Other ( )

**3. What is the number of total hospital beds?**

a. 0-100 beds

b. 101-300 beds

c. 301-500 beds

d. 501-800 beds

e. 800 or more beds

**4. What is the number of total ICU beds?**

a. 0-5 beds

b. 6-10 beds

c. 11-20 beds

d. 21-30 beds

e. 31-40 beds

f. 40 or more beds

**Note**

The following term "ICU" refers to ICU where the respondent primarily works. If the participant is involved in multiple ICUs, kindly choose one for your response.

**5. What is your ICU types?**

a. Medical-Surgical mixed ICU

b. Medical ICU

c. Surgical ICU

d. Stroke Care Unit

e. Cardiac Care Unit

f. Pediatric ICU

g. Other types of ICU

**6. Where is the primary source of ICU admission?**

a. Emergency Room

b. Wards

c. Operating room

d. Transfer from other hospital

e. Other

**7. What is the number of ICU beds? (Please provide information about the ICU you have selected.)**

beds

**8. What is characteristics of ICU?**

a. Closed ICU (Managed by intensivists)

b. Mandatory or elective critical care consultation (Between “a” and “b”)

c. Open ICU (Managed by attending physicians)

**9. What is the number of specialists in each category at your institution?**

person: Intensivist

person: Speech and language therapist

person: Certified Critical Care Nurse

person: Certified Dysphagia Nurse

**10. Are the speech and language therapist dedicated to the ICU?**

a. Yes

b. No

**11. How often does the multidisciplinary round (rounding at the bedside by the intensivist, attending physicians, pharmacists, nurses, physical therapist, or other members of the multidisciplinary team, to discuss treatment plans) occur in the ICU within a week?**

a. Not at all

b. Once a week

c. Only a week days

d. Every day

**12. Does a speech and language therapist participate in the multidisciplinary rounds in the ICU where you provide answers?**

a. Not at all

b. Occasionally

c. Every time

**13. Please provide the total number of patients for whom the speech and language therapist at your hospital conducted evaluations for dysphagia and swallowing rehabilitation on the day of the survey.**

**Note:**

**Please interview a specific speech-language therapist in your hospital.**

years: Speech and language therapist experience

patients: Total number of patients provided on the day of the survey

**14. Please provide the total number of “ICU” patients for whom the speech-language therapist at “ICU” conducted evaluations for dysphagia and swallowing rehabilitation on the day of the survey.**

**Note:**

**Please interview a specific speech-language therapist in your hospital.**

years: speech and language therapist experience

patients: Total number of patients provided on the day of the survey

**15. Do you have the following protocols implemented with multidisciplinary consensus? Please select all that apply.**

(　 ) "Pain" Protocol

( 　) "SAT" Protocol (Spontaneous Awakening Trial)

( 　) "SBT" Protocol (Spontaneous Breathing Trial)

(　 ) "Sedation" Protocol

( 　) "Delirium" Protocol

( 　) "Early Mobilization & Rehabilitation" Protocol

( 　) "Family-oriented ABCDEF Bundle, PICS Education, Promoting ICU Engagement" Protocol

(　 ) "Nutrition" Protocol

(　 ) "Physical Restraint" Protocol

( 　) "ICU Diary" Protocol

( 　) "VAP Bundle" Protocol

(　 ) "Swallowing Rehabilitation" Protocol

(　 ) None of the above

**16. What is the primary bedside screening tool for dysphagia in your ICU?**

a. Water swallowing test / modified water swallowing test

b. Repetitive Saliva Swallowing Test (RSST)

c. Food Test

d. Other　 (please specify)

**17.** **Who primarily conducts screening for dysphagia in your ICU? Please select one.**

a. Intensivist

b. Attending Physician

c. Rehabilitation Physician

d. Otolaryngologist

e. Nurse

f. Certified Nurse (Critical Care)

g. Certified Nurse (Dysphagia Care)

h. Speech and language therapist

i. Dietitian

j. Dentist

k. Dental Hygienist

l. Other

**18. What techniques are used for behavioral swallowing rehabilitation? Please select all.**

(　 ) Swallowing Exercises

(　 ) Ice Massage

(　 ) Lip closure training

(　 ) Oral motor exercise (exercises for the lips, tongue, jaw, and cheeks)

(　 ) Thermal-tactile stimulation

(　 ) Toothbrushing,

(　 ) Salivary Gland Massage

(　 ) Electrical Stimulation Therapy

(　 ) Other　 (please specify)

(　 ) Not used at all

**19. Who primarily performs behavioral swallowing rehabilitation? Please select one.**

a. Intensivist

b. Attending Physician

c. Rehabilitation Physician

d. Otolaryngologist

e. Nurse

f. Certified Nurse (Critical Care)

g. Certified Nurse (Dysphagia Care)

h. Speech and language therapist

i. Dietitian

j. Dentist

k. Dental Hygienist

l. Other

**20. What are the common reasons for difficulty in continuing swallowing rehabilitation? Please select all.**

Note: Select based on experienced cases within one month.

(　 ) Altered states of consciousness (including delirium)

(　 ) Decreased oxygen levels (hypoxia)

(　 ) Rapid breathing

(　 ) Hemodynamic instability

(　 ) Sudden arrhythmia

(　 ) High risk of aspiration/choking

(　 ) Gastrointestinal disorders such as gastrointestinal bleeding, ileus, etc.

(　 ) Inability to assume a sitting position

**21.** **Who primarily determines dietary intake and content for patients with dysphagia in the ICU.**

**Note:**

**Please choose an option that applies to more than 50% of the patients.**

a. Intensivist

b. Attending Physician

c. Rehabilitation Physician

d. Otolaryngologist

e. Nurse

f. Certified Nurse (Critical Care)

g. Certified Nurse (Dysphagia Care)

h. Speech and language therapist

i. Dietitian

j. Dentist

k. Dental Hygienist

l. Other

**22. How many average minutes per session do you conduct swallowing rehabilitation in the ICU?**

**Note:**

**Please choose an option that applies to more than 50% of the patients.**

**If swallowing rehabilitation is not conducted for more than 50% of the cases, please choose (a).**

a. 0 minutes

b. 1-5 minutes

c. 6-10 minutes

d. 11-15 minutes

e. 16-30 minutes

f. 31 minutes or more

**23. How often do you conduct swallowing rehabilitation in the ICU?**

**Note:**

**Please choose an option that applies to more than 50% of the patients.**

**If swallowing rehabilitation is not conducted for more than 50% of the cases, please choose (a).**

a. Not at all

b. Less than every other weekday and no weekends

c. Less than every other weekday and weekends

d. Every other weekday and no weekends

e. Every other weekday and weekends

f. Every weekday and no weekends

g. Every weekday and weekends

**24. Are you interested in participating in prospective studies about protocol for dysphagia in the ICU?**

a. Yes

b. No

c. Unsure

**Thank you for your participation.**
