# Supplementary material for: Prevalence and Management of Oral Intake Restrictions in Critically Ill Patients: Insights from a Multicenter Point Prevalence Study
Source: Dysphagia. 2024 Oct 21;40(4):747–58. doi: 10.1007/s00455-024-10772-5 (PMC12328520; doi:10.1007/s00455-024-10772-5)
Supplement: Supplementary file 2 — Supplementary file2 (DOCX 28 KB)—Two-day prevalence survey questionnaire [file 455_2024_10772_MOESM2_ESM.docx]

**Additional File 2**

**Survey Questionnaire**

﻿ *Incidence and Provided Care for Oral Intake Restriction in Intensive Care Unit*

You are invited to participate in a cross-sectional survey study focusing on the care of patients with oral intake restrictions in the intensive care unit (ICU). This survey has the potential to improve our understanding of dysphagia in ICUs and to establish protocols that could help provide care for dysphagia in the ICU.

This questionnaire is completely voluntary. No personally identifiable information about the patients will be collected. All data will be kept securely, and only aggregated data will be published. If you have any questions about the questionnaire, please feel free to contact the following:

This study will conduct a Point Prevalence Survey. During specific time periods (10:00 AM on November 1, 2023, and 10:00 AM on December 1, 2023), patients in the ICU will be systematically sampled to collect data.

Please review the following explanation, and if you are willing to participate in this study, check the "Agree" box at the bottom.

Important Notes:

1. If there are multiple ICUs within the hospital, please provide responses only for one ICU per respondent. (High Care Units (HCU) are not included in this survey.)

2.You need to require data collection from medical records and healthcare professionals. Since this study involves gathering information from medical records and healthcare professionals, it may take more time compared to other survey studies. To ensure the quality of the survey and consistent answers, participants were encouraged to collaborate with other healthcare professionals when completing the questionnaire. Your participation is crucial to the success of the research, despite the time and effort required. We appreciate your cooperation. Thank you very much.

Study coordinator

Takashi Hongo, MD, PhD

Department of Emergency, Critical Care, and Disaster Medicine

Faculty of Medicine, Dentistry and Pharmaceutical Sciences, Okayama University, Okayama, Japan

Email: pwup5kuf@s.okayama-u.ac.jp

Phone: 086-235-7426

**1. Are you participated in this survey?**

**a Agree**

**b Not Agree**

**2. Representative's name**

**( )**

**Important note**

1. "ICU” where the respondent provides answers ICU where the survey participant is currently associated with or primarily works. This refers to the same ICU as in the previous survey.

2. Please provide responses regarding patients who are in the ICU at the time of the survey on November 1, 2023 (1st survey) and December 1, 2023 (2nd survey), both at 10:00 AM.

**1.1. Please provide the following numbers for the patients in the ICU**

**patients**: Total number of patients in the ICU

**patients**: Patients with endotracheal intubation

**patients**: Patients with tracheostomy

**patients**: Patients extubated and liberated from mechanical ventilation in the ICU (including patients with NPPV/NHF)

**patients**: Patients admitted to the ICU due to cerebrovascular diseases

**1.2. Please provide the following numbers for the patients in the ICU**

**(Note: Please ensure that the total matches the total number of patients in the ICU)**

**patients**: 0 -19 years

**patients**: 20 - 64 years

**patients**: 65 - 74 years

**patients**: ≥75 years

**1.3. Please provide the following numbers for the patients in the ICU**

**(Note: Please ensure that the total matches the total number of patients in the ICU)**

**patients**: Male

**patients**: Female

**Section 2: Oral Intake**

2.1-2.4 Inquire about the dietary status of patients on the survey date or prior to ICU admission.

Note: (1) Select only one option that best applies to each patient. (2) Please respond based on chart information such as progress notes or dietary calendars.

We collected The Function Oral Intake Scale (FOIS) data of all patients admitted ICU.

FOIS is a scale used to assess the dietary status of patients based on their oral intake capabilities. It ranges from Level 1, indicating enteral nutrition intake only, to Level 7, which represents unrestricted oral nutrition intake

Level1. Nothing to mouth

Level2. Tube dependent with minimal attempts of food or liquid.

Level3. Tube dependent with consistent oral intake of food or liquid.

Level4. Total oral diet of a single consistency.

Level5. Total oral diet with multiple consistencies, but requiring special preparation or compen-sations.

Level6. Total oral diet with multiple consistencies without special preparation, but with specific food limitations.

Level7. Total oral diet with no restrictions.

**2.1. What is the number of patients who were able to consume oral diet with no restrictions (FOIS: Level 7) before ICU admission?**

**patients**:

**2.2. Please provide** **dietary status in the ICU on the survey date**

**(Note: Ensure the total matches the total number of patients in the ICU)**

**patients**: FOIS: Level 7

**patients**: FOIS: Levels 5 and 6

**patients**: FOIS: Levels 4

**patients**: FOIS: Levels 3 and 2

**patients**: FOIS: Levels 1 or Nil by Mouth

**2.3. Please provide** **dietary status** **of patients who have been extubated in the ICU on the survey date**

**(Note: Ensure the total matches the total number of patients extubated and liberated from mechanical ventilation in the ICU)**

**patients**: FOIS: Level 7

**patients**: FOIS: Levels 5 and 6

**patients**: FOIS: Levels 4

**patients**: FOIS: Levels 3 and 2

**patients**: FOIS: Levels 1 or Nil by Mouth

**2.4. Please provide** **dietary status** **of patients admitted to the ICU due to cerebrovascular disease on the survey date**

**(Note: Ensure the total matches the total number of patients admitted to the ICU due to cerebrovascular diseases)**

**patients**: FOIS: Level 7

**patients**: FOIS: Levels 5 and 6

**patients**: FOIS: Levels 4

**patients**: FOIS: Levels 3 and 2

**patients**: FOIS: Levels 1 or Nil by Mouth

**Section 3: Provided Care for Dysphagia**

You're almost done. Your data is invaluable, so please focus and provide your responses until the end. Please indicate the number of patients corresponding to Additional File:

**3.1. Please provide the numerical count for each item in Additional File**

**(Note: Ensure the total matches the total number of patients in the ICU)**

①( ) ②( ) ③( ) ④( ) ⑤( ) ⑥( ) ⑦( ) ⑧( ) ⑨( ) ⑩( )

⑪( ) ⑫( ) ⑬( ) ⑭( ) ⑮( ) ⑯( ) ⑰( ) ⑱( ) ⑲( ) ⑳( )

㉑( ) ㉒( ) ㉓( )

**3.2. For patients who have been extubated in the ICU, please provide the numerical count for each item in Additional File:**

**(Note: Ensure the total matches the total number of patients extubated and liberated from mechanical ventilation in the ICU)**

①( ) ②( ) ③( ) ④( ) ⑤( ) ⑥( ) ⑦( ) ⑧( ) ⑨( ) ⑩( )

⑪( ) ⑫( ) ⑬( ) ⑭( ) ⑮( ) ⑯( ) ⑰( ) ⑱( ) ⑲( ) ⑳( )

㉑( ) ㉒( ) ㉓( )

**3.3. For patients admitted to the ICU due to cerebrovascular diseases, please provide the numerical count for each item in Additional File:**

**((Note: Ensure the total matches the total number of patients admitted to the ICU due to cerebrovascular diseases)**

①( ) ②( ) ③( ) ④( ) ⑤( ) ⑥( ) ⑦( ) ⑧( ) ⑨( ) ⑩( )

⑪( ) ⑫( ) ⑬( ) ⑭( ) ⑮( ) ⑯( ) ⑰( ) ⑱( ) ⑲( ) ⑳( )

㉑( ) ㉒( ) ㉓( )

**3.4 Among the patients currently admitted to the ICU on the survey date who are suspected of having dysphagia but have not conduct swallowing screening (Figure A, Question 6), please provide the main reasons:**

( ) please specify

**Section 4: Opinion**

**4.1 If you have any opinions regarding dysphagia/speech and language therapy in the ICU, please share them:**

( ) please specify

**4.2 If there have been any complaints from patients/patient families regarding dysphagia/speech and language therapy in the ICU, please share them:**

:

( ) please specify

**Thank you for your participation.**

**Additional File**

**Please replace the placeholders (e.g., N=(〇)) with the actual numbers you have for each question.**

**Swallow Screening**

Q1. Patients who conducted swallowing screening (e.g., water swallow test) at least once between ICU admission to the survey date: N=(①)

Q2. Patients who conducted swallowing screening (e.g., water swallow test) within 24 hours after ICU admission: N=(②)

Q3. Patients for whom the initial swallowing screening (e.g., water swallow test) was conducted by a nurse between ICU admission to the survey date: N=(③)

Q4. Patients for whom swallowing screening was conducted using water swallow test/modified water swallow test: N=(④)

Q5. Patients in whom suspicion of dysphagia based on swallowing screening: N=(⑤)

Q6. Patients suspected of dysphagia but not conducted swallowing screening: N=(⑥)

**Video Fluoroscopic Swallow Study / Fiberoptic Endoscopic Evaluation of Swallowing**

Q7. Patients who underwent Video Fluoroscopic Swallow Study (VFSS) or Fiberoptic Endoscopic Evaluation of Swallowing (FEES) at least once between ICU admission to the survey date: N=(⑦)

Q8. Patients from Q5 (N=⑤) who underwent VESS or FEES between ICU admission to the survey date: N=(⑧)

**Compensatory swallowing rehabilitation**

Q9. Patients who received compensatory swallowing rehabilitation at least once between ICU admission to the survey date: N=(⑨)

Q10.11 Patients for whom the profession performing compensatory swallowing rehabilitation is a speech-language pathologist (N=(⑩)) / nurse (N=(⑪))

Q12. Number of patients from Q5 (N=⑤) who underwent compensatory swallowing rehabilitation between ICU admission to the survey date: N=(⑫)

**Behavioral swallowing rehabilitation**

Q13. Patients who received behavioral swallowing rehabilitation at least once between ICU admission to the survey date: N=(⑬)

Q14.15 Patients for whom the profession performing behavioral swallowing rehabilitation is a speech-language pathologist (N=(⑭)) / nurse (N=(⑮))

Q16. Patients from Q5 (N=⑤) who underwent behavioral swallowing rehabilitation between ICU admission to the survey date: N=(⑯)

**Oral Care / Dental Treatment**

Q17. Patients who received oral care at least once between ICU admission to the survey date: N=(⑰)

Q18. Patients from Q5 (N=⑤) who received oral care between ICU admission to the survey date: N=(⑱)

Q19. Patients who underwent dental treatment performed by a dentist at least once between ICU admission to the survey date: N=(⑲)

Q20. Patients from Q5 (N=⑤) who underwent dental treatment between ICU admission to the survey date: N=(⑳)

**Diet Modification**

Q21. Patients who underwent diet modification at least once between ICU admission to the survey date: N=(㉑)

Q22. Patients for whom diet modification was conducted during multidisciplinary rounds: N=(㉒)

Q23. Patients from Q5 (N=⑤) for whom diet modification was conducted between ICU admission to the survey date: N=(㉓)
