# Supplementary material for: Prevalence and Management of Oral Intake Restrictions in Critically Ill Patients: Insights from a Multicenter Point Prevalence Study
Source: Dysphagia. 2024 Oct 21;40(4):747–58. doi: 10.1007/s00455-024-10772-5 (PMC12328520; doi:10.1007/s00455-024-10772-5)
Supplement: Supplementary file 3 — Supplementary file3 (DOCX 20 KB) [file 455_2024_10772_MOESM3_ESM.docx]

**Additional File 3**

**Survey Questionnaire**

We are grateful for your participation in this cross-sectional survey study focusing on the care of patients with oral intake restrictions in the ICU. We have decided to conduct an additional survey on the role of dentists in the ICU, as they are considered to play a significant role. To ensure that the responses reflect the situation at the time of the original surveys conducted in 2023, we kindly ask all respondents from the initial survey to provide information based on the circumstances in 2023.

Study coordinator

Takashi Hongo, MD, PhD

Department of Emergency, Critical Care, and Disaster Medicine

Faculty of Medicine, Dentistry and Pharmaceutical Sciences, Okayama University, Okayama, Japan

Email: pwup5kuf@s.okayama-u.ac.jp

Phone: 086-235-7426

**Important note**

1. **“ICU” refers to the ICU where the respondent currently works or is primarily associated with. This is the same ICU referenced in the previous survey.**
2. **Please provide responses regarding the role of dentists in the ICU for the 2023 fiscal year (first survey conducted in November 2023).**

***ICU Dental Services***

**1.1 Are dental services provided by dentists in the ICU?**

**Yes　(　)**

**No　(　)**

**1.2 Are dentists in the ICU involved in assessment or treatment of dysphagia treatments?**

**Yes　(　)**

**No　(　)**

**1.3 Please provide information on the ICU treatments performed by dentists in the ICU, aside from those related to dysphagia.**

**(　　　　　　　　　　　　　　　　　　　　　　　　　　　　　　　　　　)**

**Thank you for your participation.**
